# Assessment of the usefulness of anti-Wb123 antibody for post-elimination surveillance of lymphatic filariasis

**DOI:** 10.1186/s13071-020-04535-y

**Published:** 2021-01-06

**Authors:** Ameyo Monique Dorkenoo, Adjaho Koba, Wemboo A. Halatoko, Minongblon Teko, Komlan Kossi, Kossi Yakpa, Rachel N. Bronzan

**Affiliations:** 1 Faculté des Sciences de la Santé, Université de Lomé, Boulevard Eyadema, 01BP 1515, Lomé, Togo; 2 Ministère de la Santé et de l’Hygiène Publique Togo, Angle Avenue Sarakawa et Avenue du 24 Janvier, 01BP 336, Lomé, Togo; 3 Institut National d’Hygiène, 1 Rue Namgbeto, Quartier administratif, 01BP 1396, Lomé, Togo; 4 Health and Development International, 8 Essex St, Newburyport, MA USA

**Keywords:** Lymphatic filariasis, Surveillance, Post-validation surveillance, Wb123, Togo

## Abstract

**Background:**

The World Health Organization has targeted lymphatic filariasis (LF) for elimination as a public health problem and recommends, among other measures, post-elimination surveillance of LF. The identification of sensitive and specific surveillance tools is therefore a research priority. The * Wuchereria bancrofti*-specific antigen Wb123-based enzyme-linked immunosorbent assay (Wb123 ELISA) detects antibodies to the recombinant Wb123 antigen of *W. bancrofti* and may be useful as a surveillance tool for LF. Six years after stopping mass drug administration to eliminate LF and recording successful results on two post-treatment transmission assessment surveys, a study was conducted in Togo aimed at helping to identify the role of the Wb123 ELISA in post-validation surveillance of LF.

**Methods:**

This was a cross-sectional study in eight previously LF-endemic districts and one non-endemic district in Togo. In each sub-district of these nine districts, two schools were selected and 15 children aged 6 to 9 years old at each school provided finger-stick blood for testing for antibodies to Wb123 using the Filaria Detect™ IgG4 ELISA kit® (InBios, International, Inc., Seattle, WA, USA).

**Results:**

A total of 2654 children aged 6 to 9 years old were tested in 134 schools in the nine districts. Overall, 4.7% (126/2654) children tested positive for antibodies to the Wb123 antigen of *W. bancrofti*. The prevalence of Wb123 antibodies varied across the eight previously endemic LF districts, from 1.56 to 6.62%. The highest prevalence, 6.99%, was found in the non-endemic district, but this was not significantly different from the average of all the LF districts (4.49%, *P* = 0.062).

**Conclusions:**

The Wb123 ELISA was positive in 4.7% of Togolese school-age children who were almost certainly unexposed to LF. This apparent lack of specificity in the Togo context makes it difficult to establish a seroprevalence threshold that could serve to signal LF resurgence in the country, precluding the use of this test for post-validation surveillance in Togo. There remains a need to develop a useful and reliable test for post-elimination surveillance for LF in humans.
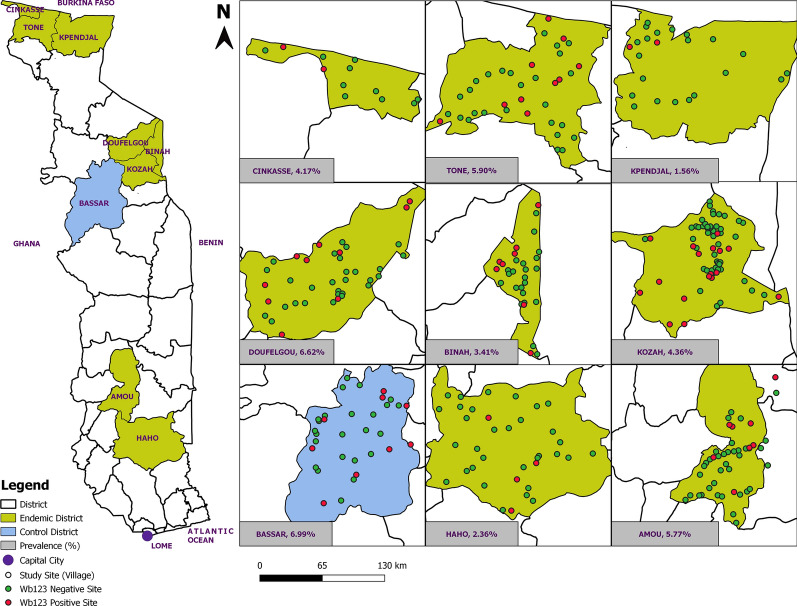

## Background

Lymphatic filariasis (LF) is a parasitic disease transmitted by several species of mosquito and caused by infection with any of three nematodes: *Wucheria bancrofti*;* Brugia malayi*; or *Brugia timori* [1]. LF is endemic in 72 countries, with an estimated 120 million people at risk, and it is one of the leading infectious causes of physical disability due to its severe complications of lymphedema (elephantiasis) and hydrocele. LF is considered to be one of the neglected tropical diseases (NTDs) for which the World Health Organization (WHO) recommends preventive chemotherapy as the primary public health intervention. The disease has been targeted for worldwide elimination [2–4].

Togo is one of 34 African countries persistently endemic for LF. In response to the WHO’s call to member states in 1997 to eliminate LF, Togo launched its National Program for the Elimination of Lymphatic Filariasis (NPELF) and completed surveys and studies that identified eight of its 40 districts as being endemic for the disease. From 2000 to 2009, mass drug administration (MDA) was initiated among eligible populations in each endemic district for a period of at least 5 years. Following the completion of successful transmission assessment surveys (TAS) in 2009, MDA was stopped in 2010 [5–7].

In accordance with WHO recommendations, Togo subsequently conducted two additional rounds of TAS in 2012 and 2015, respectively, during which the prevalence of an antigen specific to *W. bancrofti* in children born since the MDA activities was measured. The two post-treatment TAS successfully demonstrated that the prevalence of *W. bancrofti* antigen in these children, measured 3 and 6 years after the last MDA, was well below 2%, the threshold below which LF transmission should be successfully interrupted [8]. In addition to these two TAS, Togo conducted other surveillance activities between 2010 and 2016 using the Og4C3 enzyme-linked immunosorbent assay (ELISA), filariasis test strips (FTS) and thick blood films; no evidence of LF transmission was found [9–11].

Despite the apparent success of the TAS, the tests used are limited by their sensitivity, specificity and/or the long latency period between infection with the parasite and the development of a positive test result. Togo is bordered by three LF-endemic countries, namely Benin, Ghana and Burkina Faso, which poses a risk for re-introduction of the disease in Togo. Consequently, there is a need for an effective surveillance tool [12].

As more countries are nearing completion of their LF MDA programs, the identification of sensitive and specific surveillance tools has become a research priority. The* W. bancrofti*-specific antigen Wb123-based ELISA (Wb123 ELISA) detects antibodies to the recombinant Wb123 antigen of *W. bancrofti*, an early and specific marker of *W. bancrofti* infection, and thus may be useful as a surveillance tool for LF in the post-treatment setting [13, 14].

While preparing to submit its application to the WHO for validation of elimination of LF as a public health problem, and as a means of corroborating post-treatment surveillance data, a study of the prevalence of anti-Wb123 IgG4 antibodies among children in the eight districts previously endemic for LF was conducted in Togo. The objective of the study reported here was to help identify the role of the Wb123 ELISA in post-validation surveillance of lymphatic filariasis.

## Methods

### Study design

This was a cross-sectional, school-based survey carried out from 15 February to 31 March 2015. We used the platform of a national survey of the impact of MDA on the control of soil-transmitted helminths (STH) and schistosomiasis in Togo to measure the seroprevalence of antibodies to the Wb123 antigen of *W. bancrofti* in school-going children [15].

### Sampling

#### Selection of survey sites

**Fig. 1:**
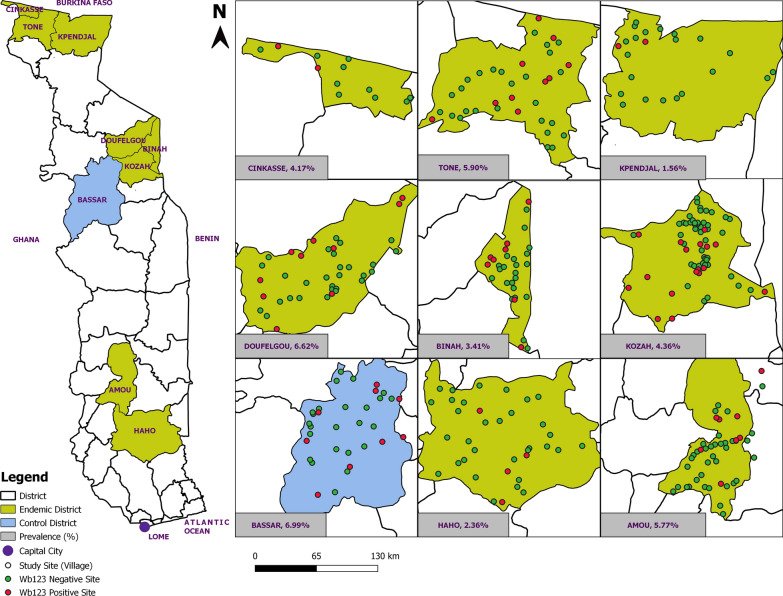
Distribution of the surveyed schools (circles) and sites where at least one child tested positive for antibodies to * Wuchereria bancrofti*-specific antigen Wb123 (red circles)

The MDA impact survey, conducted by the Ministry of Health (MOH) and described in detail elsewhere, used the same methodology as had been used for the baseline prevalence survey of STH and schistosomiasis in 2009 [15, 16]. For the present nested study of Wb123 IgG4 seroprevalence, nine of the 35 districts surveyed for STH/schistosomiasis were included, namely the eight districts previously endemic for LF (Amou, Binah, Cinkassé, Doufelgou, Haho, Kozah, Kpendjal and Tone) and the district of Bassar, which has never been endemic for LF and was used as a negative control for this survey (Fig. 1). In every sub-district of each of the nine districts, the same two villages surveyed in 2009 were revisited, and sampling was carried out at the public schools that had been included in the baseline survey.

#### Sampling of children

At each school, children aged between 6 and 9 years (equivalent of first or second grade) participated in the survey. To assess the impact of the MDA, the day before the survey, a convenience sample of 30 children was selected by the headmaster or a teacher at each school, and a consent form was sent home to each child’s guardian. On the day of the study, the first 15 children who had received written parental consent and were able to provide both urine and stool sample were enrolled in the impact assessment. In the nine districts selected for this Wb123 study, the first eight of these 15 children were enrolled for testing by Wb123 ELISA.

First, 60 μl of finger-stick capillary blood was collected from each participating child and placed on the six round protuberances of a filter paper disk (designed to hold 10 µl of whole blood per tab) (TropBio; Cellabs Diagnostics Pty Ltd, Brookvale, Sydney, Australia). After being fully air dried, each filter paper sample was placed in an individual plastic bag with a desiccant sachet, sealed and stored at ambient temperature until delivery to Togo’s National Institute of Hygiene (Institut National d’Hygiène [INH] Lomé, Togo), where they were stored at 4 °C. Each sample was tested for IgG4 anti-Wb123 antibodies, specific to *W. bancrofti*, using the Filaria Detect™ IgG4 ELISA kit® (InBios; International, Inc., Seattle, WA USA).

### Laboratory testing

Samples were processed using the ELISA according to the manufacturer’s instructions. From each filter paper, one tab with 10 µl of dried blood was incubated overnight at 4 °C with 250 µl of sample buffer to obtain the eluate. Then, 100 µl of eluate from each sample was placed in each of two wells of the microtiter plate pre-coated with Wb123 antigen. Controls included two wells of 100 µl of each the three controls supplied in the kit (positive, weak positive and negative) and two wells of 100 µl each of two positive controls (H3, high-positive control; H19, low-positive control) supplied by the U.S. Centers for Disease Control (CDC; Atlanta, GA, USA). The plates were incubated 30 min at 37 °C and washed six times. Anti-human IgG4 antibody conjugated with enzymatic horseradish peroxidase (HRP) was added to the wells and the plates incubated again at 37 °C for an additional 30 min. After a second series of six washes, the wells were incubated with tetramethylbenzidine (TMB) at room temperature for 15 min. The reaction was stopped by the addition of 50 µl of 1N sulfuric acid.

The plates were read at 450 nm using a microplate reader (Biotek® ELx800®; Biotek Instruments, Inc., Winooski, VT, USA). The average of the optical densities (OD) from each pair of duplicate wells was used in the final analysis.

The following criteria were used to evaluate the quality of each plate; the mean OD of the negative control should be < 0.200; the mean OD of the positive control should be > 0.500; the mean OD of the low-positive control should be greater than the mean OD of the negative control; the mean OD of the H3 control must be between 0.954 and 1.315; the mean OD of the H19 control must be between 0.396 and 0.542; and the coefficient of variation (CV) of each duplicate pair of samples (the standard deviation of each sample from the pair’s mean, divided by the mean) must be < 20%.

A Gaussian mixture model was fitted to the data to determine the cut-offs for positive, negative and indeterminate results [17, 18]. The mean OD values were first normalized to the CDC H19 control to reduce plate-to-plate variability, and an expectation maximization (EM) algorithm was run using all samples with a CV < 20%. The optimal fit was a skew-normal distribution with four components, as determined by the lowest Bayesian Information Criterion. Based on the H19 OD cut-offs reported for São Tomé, Chad and Gabon (0.49, 0.48 and 0.41, respectively; Katie Gass and Sarah Sullivan, personal communication), we elected to set the cut-off for positive samples between the second and third sub-populations—in this case, an OD of 0.50. We added an uncertainty range around that 0.50 cut-off, within which the certainty of classification of those OD values is < 80%. The lower bound of this uncertainty range, 0.44, was the cut-off for negative samples, the upper bound, 0.57, was the cut-off for positive samples and any values lying within the range 0.44 to 0.57 were considered indeterminate results. We secondarily analyzed the results using a cut-off set between the third and fourth sub-populations of the distribution.

### Data management and analysis

The data were entered into a database designed with Epi Info™ version 3.5.2 (CDC; Atlanta, GA, U.S) and analyzed with SPSS Statistics® (IBM Corp. Armonk, NY, USA) and Stata 13.1 (StataCorp, College Station, TX, USA). Proportions were compared using Chi-square or the Fisher exact tests. The threshold of significance was set at α = 5% for all tests performed.

### Ethical considerations

The protocol for this study was approved by the Bioethics Committee for Health Research of the Togo MOH. In addition, written informed consent was obtained from the parents or guardian of each child enrolled in this study.

## Results

A total of 2654 children aged between 6 and 9 years were tested in 134 schools in the nine districts. The characteristics of the surveyed population are shown in Table 1.

**Table 1:** Characteristics of the study population

Region	District	Number of rounds of MDA received	Numbers surveyed^a^						
			Total *N* (%)	Sex,		Age (years)			
				Female	Male	6	7	8	9
Savanes	Kpendjal	8	192	82 (42.7)	110 (57.3)	0 (0.0)	3 (1.6)	66 (34.4)	123 (64.1)
	Cinkassé	9	96	55 (57.3)	41 (42.7)	4 (4.2)	23 (24.0)	40 (41.7)	29 (30.2)
	Tone	9	288	126 (43.8)	162 (56.3)	5 (1.7)	60 (20.8)	106 (36.8)	117 (40.6)
Kara	Doufelgou	8	302	131 (43.4)	171 (56.6)	2 (0.7)	39 (12.9)	117 (38.7)	144 (47.7)
	Binah	8	264	123 (46.6)	141 (53.4)	7 (2.7)	37 (14.0)	85 (69.1)	135 (51.1)
	Kozah	6	528	258 (48.9)	270 (51.1)	23 (4.4)	82 (15.5)	173 (32.8)	250 (47.3)
Plateaux	Amou	7	416	205 (49.3)	211 (50.7)	6 (1.4)	51 (12.3)	111 (26.7)	248 (59.6)
	Haho	7	296	113 (38.2)	183 (61.8)	1 (0.3)	62 (20.9)	118 (39.9)	115 (38.9)
Total for endemic districts			2382	1093 (45.9)	1289 (54.1)	48 (2.0)	357 (15.0)	816 (34.3)	1161 (48.7)
Bassar (control)		Not applicable	272	126 (46.3)	146 (53.7)	18 (6.6)	55 (20.2)	101 (37.1)	98 (36.0)
Total surveyed			2654	1219 (45.9)	1435 (54.1)	66 (2.5)	412 (15.5)	917 (34.6)	1259 (47.4)

MDA, Mass drug administration

^a^Values are presented as the number (*N*) of children with the percentage in parentheses

Overall, 4.7% (126/2654) children tested positive for antibodies to the Wb123 antigen of *W. bancrofti*, and 1.92% (51/2654) children had indeterminate results. The prevalence of Wb123 antibodies varied across the eight previously endemic LF districts, from a low of 1.56% in Kpendjal to a high of 6.62% in Doufelgou (Fig. 1). The highest prevalence was found in the non-endemic district of Bassar (6.99%), but this was not significantly different from the average of all the LF districts (4.49%, *P  *= 0.062, Table 2).

**Table 2:** Prevalence of anti-Wb123 IgG4 antibodies by district, age and gender and number of rounds of mass drug administration with ivermectin and albendazole

Characteristics		Seroprevalence						*p* value
		Total	Indeterminate			Positive		
			*N*		(%)	*N*	%	
Region	District							
Savanes	Kpendjal	192	2		1.04	3	1.56	0.001
	Cinkassé	96	4		4.17	4	4.17	
	Tone	288	3		1.04	17	5.90	
Kara	Doufelgou	302	6		1.99	20	6.62	
	Binah	264	4		1.52	9	3.41	
	Kozah	528	12		2.27	23	4.36	
Plateaux	Amou	416	12		2.88	24	5.77	
	Haho	296	1		0.34	7	2.36	
Type of district								
All districts		2654	51	1.92		126	4.75	
All endemic districts		2382	44	1.85		107	4.49	0.062
Bassar (Control)		272	7	2.57		19	6.99	
Age (years)								
6		66	1	1.52		3	4.55	0.412
7		412	3	0.73		21	5.10	
8		917	15	1.64		39	4.25	
9		1259	32	2.54		63	5.00	
Gender								
Girls		1219	19	1.56		41	3.36	0.002
Boys		1435	32	2.23		85	5.92	
Number of rounds of MDA								
No MDA (non-endemic)		272	7	2.57		19	6.99	0.905
6 rounds		528	12	2.27		23	4.36	
7 rounds		712	13	1.83		31	4.35	
8 rounds		758	12	1.58		32	4.22	
9 rounds		384	7	1.82		21	5.47	

Boys were significantly more likely to have antibodies to Wb123 than were girls (5.92* vs* 3.36%, respectively; *P* = 0.002). There was no significant difference in seroprevalence by age of the children or number of rounds of MDA for LF received (*P* = 0.412 and *P* = 0.905, respectively; Table 2).

If we set the cut-off for positive and negative samples between the third and fourth sub-populations of the distribution (without adding an uncertainty range around that cut-off), then only 0.77% of the samples would fall above that raw cut-off.

## Discussion

Antibody testing for lymphatic filariasis has been proposed for post-validation surveillance of LF. In the present study, we measured the prevalence of antibodies to the Wb123 antigen of *W. bancrofti* in school-age children in Togo 6 years after the last MDA for LF. The prevalence of Wb123 antibodies was 4.5% across all districts previously endemic for LF and 7% in a non-endemic control district.

In April 2017, Togo was recognized by the WHO as the first sub-Saharan country to have eliminated LF [19]. In addition to MDA, Togo’s achievement was also due to the presence of an efficient, nation-wide laboratory and clinic-based surveillance system, established in 2006 before the end of MDA, as well as information from two TAS carried out in 2012 and 2015 that were recommended by WHO [7, 9, 20]. Continued surveillance for LF will be critical to preserving Togo’s success.

The children in this survey were born shortly before or after MDA for LF was discontinued in 2009. Extensive surveillance for LF in Togo from 2010 through to the time of this study in 2015 showed no evidence of LF transmission in Togo. Over the course of those 6 years, more than 26,000 people were screened by nocturnal thick blood smear for microfilaria, and only one positive was found. Another 6788 people were tested by the Og4C3 ELISA; of the 13 people testing positive, none was positive for microfilaremia by nocturnal thick blood smear [10]. Additionally, Togo achieved excellent results in the TAS carried out in 2012 and 2015; in both years, more than 6000 children in the same age group and same districts as this study were tested by the immunochromatographic card test or FTS [8, 10]. The 2015 TAS was conducted approximately 4 weeks before the present study; 6 children tested positive by FTS and only three of these tested positive for microfilaria by the nocturnal smear. In the 2012 TAS, 13 children were antigen positive, and no child was positive for microfilaria. Togo has also continued annual mass door-to-door distribution of ivermectin (to all people aged 5 years and older, for onchocerciasis) and albendazole (to school-age children, for STH) from 2010 through to the present in these same previously LF-endemic districts. Although we did not collect data on the history of residence outside of Togo’s borders, we believe it is unlikely that imported infections could account for the high prevalence of Wb123 antibodies observed in this post-elimination setting. Taken together, these data indicate that the children in this study had essentially never been exposed to LF transmission in their lifetime. Thus, the positive Wb123 results likely reflect the imperfect specificity inherent to antibody-based assays. Such a high prevalence of antibodies in a setting where LF has been eliminated as a public health problem suggests that the Wb123 ELISA is unlikely to be a useful tool for post-elimination surveillance of LF.

In the LF post-validation elimination setting, which is where Togo is at the present time, it is important to have simple, sensitive, specific and commercially available tools for detecting *W. bancrofti* infection. Four types of diagnostic tools are currently available for monitoring the impact of MDA, including: nocturnal blood smear for microfilaraemia; *W. bancrofti* antigen detection tests; filarial antibody detection tests; and PCR techniques for the detection of filarial infection [21–25]. However, smears for microfilaria suffer from poor sensitivity, antigen tests may take months to become positive after infection and PCR techniques have not proven to be an efficient diagnostic method.

In recent years there has been growing interest in antibody-based assays that can detect exposure to third-stage larva (L3), such as assays for the Wb123 antibody, as these allow early detection of potential *W. bancrofti* transmission [14]. Antifilarial antibody responses can serve as an important epidemiological indicator in a sentinel population of young children and thus may be valuable as tool for surveillance in the context of LF elimination programs [26]. Although some antibody tests have not met the requirement for specificity in regions of co-endemicity with other filarial infections, as in Togo, the Wb123 ELISA has proven to be both sensitive and specific. Hamlin et al. [26] demonstrated that antifilarial antibodies are early markers of infection and that they develop before circulating filarial antigen. Other studies have demonstrated an increase in antibody prevalence to Wb123 in a longitudinal cohort of children as well as a decrease in Wb123 antibody prevalence in response to efforts to reduce LF transmission [26–28]. The study presented here was conducted to assess whether the Wb123 ELISA can be used in the post-validation setting of LF elimination in Togo.

An antibody-based assay will only be a good early indicator of resurgence of infection if unexposed children are essentially all negative based on the ELISA results—and not if the prevalence of antibody in a population of unexposed children is as high as that found in this study. In addition to the 4.7% of children who tested positive for Wb123 antibody, a non-negligible proportion (1.85%) of the children tested had indeterminate results as determined by the mixture model. Won et al. [29] rightly suggest that the Wb123 ELISA might be useful for demonstrating interruption of transmission, although there is currently no established antibody threshold to indicate interruption of transmission. However, a test designed for early and accurate identification of renewed LF transmission in a post-elimination setting would need to be sufficiently specific to be able to detect small increases in antibody prevalence to provide a warning signal before a putative threshold for transmission was exceeded.

A specific challenge of this assay is to establish the cut-off value for positive and negative results. Instructions in the Wb123 ELISA kit indicate that the cut-off should be determined by testing a minimum of 100 specimens from three categories of individuals from the geographic area where the kit is being used: individuals with known filarial-positive LF; individuals with confirmed related parasitic diseases or unrelated febrile diseases (e.g. *Onchocerca volvulus* or malaria); and 100 normal healthy adults. In an LF post-elimination setting as found in Togo, it is not possible to identify 100 people with proven, filarial-positive disease. To circumvent this problem, we fit the data to a Gaussian mixture model to determine the cut-offs for positive, negative and indeterminate results, respectively. This method poses its own challenges, as the correct cut-off must be inferred from past experience if there are more than two components to the model. In the case of Togo, if a higher cut-off for positives were used, for example if the cut-off were set between the third and fourth populations in the distribution, 0.77% of the population would fall above the crude positive cut-off. In Mali, researchers found less than 1% prevalence of anti-Wb123 antibodies in a similar population, suggesting that perhaps the lower (0.77%) prevalence would be a better choice of cut-off in Togo, but even this cut-off would yield too many positives to be of use in a post-elimination setting [30]. This difficulty in establishing an appropriate cut-off is a key challenge that limits the utility of the Wb123 ELISA as a stand-alone tool for LF surveillance, particularly in a low- prevalence setting.

Others have noted the challenges of interpreting the results of the Wb123 assay in a post-treatment setting, and that the correct determination of robust cut-offs is essential to the practical application of antibody assays for LF [31, 32]. Many studies in the literature have calculated cut-off values for the Wb123 ELISA from receiver operating characteristic (ROC) curves using well-characterized panels of samples [13, 26, 29, 33]. This approach may account for the lower prevalence of Wb123 observed in some of these studies in comparison with the mixture model used in similar populations in the current study. However, ROC curves would ideally employ well-characterized panels of samples from the region where the ELISA is being employed to account for regional differences in the prevalence of related parasitic diseases and in host genetics. As already mentioned, such panels of locally obtained LF microfilaria-positive samples cannot be constructed in countries that have already eliminated LF. The absence of standardized control samples for establishing clear cut-offs limits the utility and interpretability of this assay.

## Conclusions

Postive results for the Wb123 ELISA were obtained in 4.7% of Togolese school-age children who were almost certainly unexposed to LF. The apparent lack of specificity in the Togo context makes it difficult to establish a seroprevalence level that could serve to signal LF resurgence in the country, precluding the use of this test for post-validation surveillance in Togo. Practical application of this test for post-elimination surveillance in any setting would require additional work to determine cut-off values appropriate for the local context. Although the Wb123 ELISA represents a significant improvement in specificity over many other LF antibody tests, there remains a need to develop a useful and reliable test for post-elimination surveillance for LF in humans.

## Data Availability

The datasets used and analyzed during the current study are available from the corresponding author upon reasonable request.

## Abbreviations

ELISA: Enzyme-linked immunosorbent assay
EM: Expectation maximization
FTS: Filariasis test strip
HRP: Horseradish peroxidase
LF: Lymphatic filariasis
MDA: Mass drug administration
MOH: Ministry of Health
NPELF: National Program for Elimination of Lymphatic Filariasis
NTD: Neglected tropical diseases
STH: Soil-transmitted helminths
TAS: Transmission assessment survey
TMB: Tetramethyl benzidine
WHO: World Health Organization
